# An exploration of the data collection methods utilised with children, teenagers and young people (CTYPs)

**DOI:** 10.1186/s13104-015-1018-y

**Published:** 2015-03-01

**Authors:** Sarah M Flanagan, Sheila Greenfield, Jane Coad, Susan Neilson

**Affiliations:** The Department of Primary Care Clinical Sciences, The University of Birmingham, B15 2TT Edgbaston, Birmingham, UK; Centre for Children and Families Applied Research, Faculty of Health and Life Sciences, Coventry University, Priory Street, CV1 5FB Coventry, UK

**Keywords:** Children, Teenagers, Young adults, Research methods, Data collection methods

## Abstract

**Background:**

The impact of cancer upon children, teenagers and young people can be profound. Research has been undertaken to explore the impacts upon children, teenagers and young people with cancer, but little is known about how researchers can ‘best’ engage with this group to explore their experiences. This review paper provides an overview of the utility of data collection methods employed when undertaking research with children, teenagers and young people.

A systematic review of relevant databases was undertaken utilising the search terms ‘young people’, ‘young adult’, ‘adolescent’ and ‘data collection methods’. The full-text of the papers that were deemed eligible from the title and abstract were accessed and following discussion within the research team, thirty papers were included.

**Findings:**

Due to the heterogeneity in terms of the scope of the papers identified the following data collections methods were included in the results section. Three of the papers identified provided an overview of data collection methods utilised with this population and the remaining twenty seven papers covered the following data collection methods: Digital technologies; art based research; comparing the use of ‘paper and pencil’ research with web-based technologies, the use of games; the use of a specific communication tool; questionnaires and interviews; focus groups and telephone interviews/questionnaires.

The strengths and limitations of the range of data collection methods included are discussed drawing upon such issues as of the appropriateness of particular methods for particular age groups, or the most appropriate method to employ when exploring a particularly sensitive topic area.

**Conclusions:**

There are a number of data collection methods utilised to undertaken research with children, teenagers and young adults. This review provides a summary of the current available evidence and an overview of the strengths and limitations of data collection methods employed.

## Background

Each year in the UK 2,022 people aged 15-24 and 1,578 people aged 0-14 are diagnosed with cancer [1]. Although cancer is relatively uncommon in the 15-24 age groups with less than one percent of total cancers diagnosed in teenager and young people [2], it is the commonest disease related cause of death in England [3]. Furthermore, there is evidence that the incidence of cancer amongst young people is increasing, rising at an average of 0.9% per year [4]. The impact of the disease upon patients, particularly on teenagers and young people can be profound. Aside from the physical pain, changes to functional ability, social limitations, issues of an existential nature and of self-identity that accompany a cancer diagnosis and experience of the disease, teenagers and young people with cancer also experience the ‘intersection of the cancer experience with developmental tasks associated with this period’ [5]. The physical changes that occur at adolescence along with the development of a sexual identity, separation from parents and increased involvement with peers and autonomous decision making bring with them, their own particular challenges without the added trauma of experiencing the effects of a chronic and potentially life-limiting disease [6]. Furthermore, experiences of relapsed cancer can heighten the psychological burden for young people. Interviews undertaken with teenagers and young people whose cancer had returned [7] describe the sense of devastation and shock experienced on hearing the news of cancer recurrence.

The experiences of being diagnosed with, treated for, and in particular living with cancer in teenagers and young people have been explored. Cancer in this group has been described as being connected to the fear of the unknown [8] fear of alienation and of altered appearance [9,10] and fear of dying [9].

Given the physical and psychological impacts of living with cancer along with the need for researchers to engage with this group and for their voices to be heard, this paper aims to review the kinds of data collection methods preferred by teenagers and young people.

Undertaking research with teenagers, young people and adults who have been diagnosed with cancer can be problematic. There may be concerns that is it overly burdensome for individuals to participate in research whilst undergoing treatment, along with ethical issues like consent taking and family worries around fears of researchers raising potentially distressing topics

### Teenagers, young people and adolescents - terms of reference

Before undertaking the review, it was important that we were able to focus upon studies undertaken with the population of interest and it is noted that the terms ‘teenager’ and ‘young people’ are commonly synonymised with terms such as ‘adolescent’ or ‘young adult’. Some authors use the term ‘children’ if they are referring to individuals under 16 and for this reason, some of the included studies refer to participants as children rather than teenagers.

The period of adolescence is defined as ‘the period of life beginning with the appearance of secondary sexual characteristics and terminating with the cessation of somatic growth’ and although there is no general consensus about the age range defined as adolescent, it is cited as covering the ages 11 to 19. Dashiff and colleagues urge researchers to be mindful of the areas of overlap that can occur between the biological, cognitive and emotional developmental phases of adolescence, along with the fact that boys generally begin puberty one and a half to two years later than girls [11]. The research setting itself can have an effect upon how a young person responds to research questions, for example, young people’s responses in a focus group undertaken in a school setting may be influenced by peer pressure. At home, family norms may impact upon responses given by young person. Cultural norms with particular adolescent sub groups may also come into play.

Turner-Henson also notes the variation that comes with the stages of maturation in terms of adolescence in terms of biology and cognition and provides a typography of ‘adolescence’ in terms of age ranges. However, broadly speaking ‘early adolescence’ includes ages 10 to 14; middle adolescence ages 15-17 and finally, late adolescence is typified by ages 18 to 21. In terms of the development of cognitive skills, it is noted that particularly for those in early and middle adolescence, that individuals can feel that they lack authority or control in a health care setting, and may feel they have little autonomy over decisions made about their treatment [12]. Such feelings can also be applied to how they respond to research, deferring to the researcher, parents, or healthcare professionals. Such factors must be borne in mind when undertaking research, ensuring a balance is struck between scientific responsibility and participant welfare.

However, it is noted that the use of this age demarcation brings its own inherent difficulties given the variation of maturation on an individual level. A 14 year old considered to be in the early adolescent stage may be biologically, emotional and cognitively closer to a 17 year old depending on their past experiences and environmental factors.

Furthermore, adolescents can be a vulnerable group. It is important that they are active participants in the decision making process in terms of participating in research e.g. assent/consent process; knowledge of the risks and benefits of participation. Researchers must be mindful of and accountable for the power they hold in influencing decisions to participate in research.

### Ethical considerations of research with adolescents

However, there are also inherent ethical and methodological issues to be considered in research with all teenagers, young people and adolescents irrespective of the topic being explored. Clear judgment and expertise are required by researchers in ensuring that participants have been able to give fully informed consent and that participants have fully understood what they are agreeing to take part in. Furthermore, careful consideration needs to be given to the most appropriate method of data collection utilised for this group. Is a group setting appropriate for eliciting responses from teenagers and young people when the topic of interest is of a sensitive nature? Would implicit power differentials between researcher and young person impede the elicitation of meaningful or truthful responses?

It is acknowledged that medical research involving under 16 s is an important means of ‘promoting child health and well-being’ [13]. The law relating to research on children (children are in this context, defined as under 18) is not entirely clear. However, the application of general principles indicates that where children have ‘sufficient understanding and intelligence to understand what is proposed’, it is they and not their parents whose consent is required by law.

Informed consent is ‘ the process of agreeing to take part in a study based on access to all relevant and easily digestible information about what participation means, in particular in terms of harms and benefits [14].

Given the complex emotional and cognitive changes that occur during adolescence and young adulthood, ensuring that consent is viewed as a process, with the researcher constantly checking understanding and ensuring transparency and clarity in terms of the aims, objectives, risk and benefits is of particular importance.

The Society for Adolescent Medicine 2003 (USA) produced guidelines the aims of which are to ‘protect individual adolescent subjects’ [15]. These guidelines offer some pertinent considerations; for example, respecting the young person will require balancing the respect for the ‘emerging capacity’ of an adolescent for independent decision making with an awareness for that ‘special protection’ should be in place to acknowledge ‘limited cognitive capacity’ where this is deemed necessary. The past exclusions of adolescents from research has, it is noted, had a detrimental effect upon this group, who may be excluded from the benefits of participation - not having their voices heard has led to interventions or programmes that do not take into account the specific needs of this group.

As a precursor to a study to ask teenagers and young people about the data collection methods that may ‘best’ enable them to record and communicate their experiences of living with cancer a narrative review was undertaken. The review examined the literature pertaining to methods employed with teenagers, young people and adolescents irrespective of discipline e.g. education, sociology, health research, to explore the range and utility of the methods employed.

Conventional methods of data collection include questionnaires, one to one interviews and focus groups. However, in recent years, more novel methods of data collection have been trialled for use with CTYPs in a variety of medical or non-medical settings, for example the use of participatory methods [16] or digital technologies [17]. The use of video diaries as a way for CTYPs to record and communicate their experiences have been utilised [18]. The use of art and photography based techniques have also proved successful with younger children [19].

This review aims to provide a summary of the papers that discuss the utility of particular data collection methods with CTYPs.

Following initial scoping searches, it became apparent that there is paucity in the literature pertaining to the types of data collection methods utilised with CTYPs with cancer. Given this, a decision was made by the research team to widen the search to include all studies pertaining to data collection methods utilised with CTYPs.

Database searches were undertaken by an information specialist and to ensure that a systematic search of the available evidence was undertaken, the following databases were searched: Medline, Embase, Cochrane; Assia, SSCI, CINAHL, CLib, ERIC and Medline in Process (Table 1). These databases were searched to ensure that the breadth of literature across the social and medical sciences was captured. Further searches of the grey literature were undertaken to capture studies that were not returned via the database searches above.

**Table 1:** **Searched databases and results returned**

**Datebase**	**Results returned**
Medline	50
Embase	50
Cochrane	3
Assia	45
SSCI	64
CINAHL	56
CLib	90
ERIC	23
Medline in Process	3
**Total**	**384**

The search terms were: Young people, young adult, teenager, adolescent, data collection methods (any type of data collection was included to ensure the range of methods could be captured).

### Inclusion criteria

Given the heterogeneity in terms of how teenagers and young people are defined in the literature, for the purposes of this review, all studies that included data collection methods with people between the ages of 13 and 24 were included (this age range represents the patients treated on Teenager Cancer Trust units that will be recruited for the second stage of the study). All databases were searched from inception to February 2013. This enabled a wider range of studies to be included. Some studies were included that described their participants as children if the age fell within the 13-24 range. The search included national and international studies.

### Exclusion criteria

Papers where the focus was upon the study outcomes alone, rather than the data collection method per se.

The title and abstracts were imported into Reference Manager (version 12). Following removal of the duplicates, the title and abstracts of the remaining studies were assessed to check eligibility.

**Figure 1:**
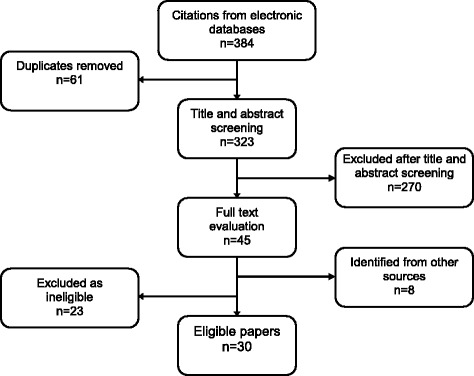
**PRISMA diagram.**

The full-text of the papers that were deemed eligible from the title and abstract were accessed and those that did not fit the eligibility criteria excluded from the study. 13 studies were included that were identified from citation searches and discussions within the research team. See Figure 1 for details of the search results.

## Findings

Due to the heterogeneity in terms of the scope of the papers identified and for ease of assessing the included papers, where possible, studies that focused upon a particular method of data collection are discussed together.

Broadly speaking, the types of data collection methods assessed in the included studies were:

- Digital technologies
- Art based research
- Comparing the use of ‘paper and pencil’ research with Web-based technologies
- The use of games
- Use of a specific communication tool
- Questionnaires and interviews
- Focus groups
- Telephone

Three papers covered a number of data collection methods and these are presented at the beginning of the review. Table 1 lists the first author, title, year of publication, country of origin, age range of population and discipline for each paper included in the review. The majority of the papers were published between 2001 and 2011, with only one paper published in 1992. There were 14 papers published in the USA; 8 from the UK; 3 from Australia and New Zealand; 3 from Europe and one paper was published in Brazil. The health disciplines were the most common source of research, with education and the social sciences also featuring in the list of identified studies. See Table 2 for the types of data collection methods identified in the review. Table 3 presents the strengths and limitations of the data collection methods studied.

**Table 2:** **Types of data collection methods identified in review**

**Data collection method**	**Title, Author**	**Country**	**Year**	**Sample size (if applicable)**	**Age range (if applicable)**	**Discipline**
**Overview of methods**	Walker S. Consulting with children and young people.	UK	2001	n/a	n/a	Social studies
	Fargas-Malet M et al. Research with children: methodological issues and innovative techniques.	UK	2010	n/a	n/a	Childhood research
	Christian BJ et al. It’s a small, small world: Data collection strategies for research with children and adolescents.		2010	n/a		Paediatric Nursing
**Digital technologies**	Murthy D. Digital Ethnography: An examination of the use of new technologies for social research.	USA	2008	n/a	n/a	Sociology
	Cranmer S. Listening to excluded young people’s perspectives on how digital technologies support and challenge their lives.	UK	2010	n=13	12-15	Education
	Baer A et al. Obtaining sensitive data through the web: An example of design and methods.	USA	2002	n=500	18-20	Health/Epidemiology
	Cleary M, Walter G. Is e-mail communication a feasible method to interview young people with mental health problems.	Australia	2011	n/a	n/a	Health/nursing
	Blackstone MM et al. Feasibility of an interactive voice response tool for adolescent assault victims.	USA	2009	n=131	12-19	Health/emergency medicine
	Trapl ES et. Use of audio-enhanced personal digital assistants for school-based data collection.	USA	2005	n=645	12-13	Health/adolescent health
	Denny SJ et al. Hand-held internet tablets for school-based data collection.	New Zealand	2008	n=177	12-17	Health
	Des Jarlais DC et al. The use of electronic debit cards in longitudinal data collection with geographically mobile drug users.	USA	2005	n=139	16-32	Health/Substance use
	Mangunkusumo RT et al. Internet- administered health questionnaires compared with a paper version in a randomized study.	Holland	2005	n=565	13-17	Health
	McCabe SE et al. Feasibility study for collecting alcohol and other drug use data among secondary school students: A web-based survey approach.	USA	2004	n=1536	11-16	Health/Substance use
	Tates K et al. Online focus groups as a tool to collect data in hard-to-include populations: examples from paediatric oncology.	Holland	2009	n=25	8-17	Health/paediatrics
**Focus Groups**	Banister E. Data collection strategies for accessing adolescent women’s worlds. 2002	Canada	2002	n=31	14-16	Health/Nursing
	Yonekura T et al. The educative game as a sensitization strategy for the collection of data with adolescents.	Brazil	2010	n=209	15-19	Education/Health
**Paper versus Computer**	Beebe T et al. The effects of data collection mode and disclosure on adolescent reporting of health behaviour.	USA	2006	n=610	12-18	Health
	Scott-Johnson PE et al. Web-based data collection: An effective strategy for increasing African Americans’ participation in health- related research.	USA	2010	n=192	18-28	Health
	Wu Y& Newfield SA. Comparing data collected by computerized and written surveys for adolescence health research. Journal of School Health	USA	2007	n=1131	12-16	Health/adolescent health/Education
	Wyrick DL& Bond L. Reducing sensitive survey response bias in research on adolescents: A comparison of Web-based and paper-and-pencil administration. American Journal of Health Promotion.	USA	2011	n=628	Unspecified (middle and high school)	Health
**Questionnaire/Interviews**	Plummer ML, et al. “A bit more truthful”: the validity of adolescent sexual behaviour data collected in rural northern Tanzania using five methods. Sex Transm Infect	UK	2004	n=9280	Mean age 15.5 years	Health
	Dockrell J, Joffe H. Methodological issues involved in the study of young people and HIV/AIDS: a social psychologicial view. Health Education Research.	UK	1992	n/a	Not defined (young people)	Health and Education
	Kann Let al As assessment of the effect of data collection setting on the prevalence of health risk behaviours among adolescents. Journal of Adolescent Health.	USA	2002	Unclear	14-17	Health
**Telephone**	Ellen JM et al. A randomized comparison of A-CASI and phone interviews to assess STD/HIV-related risk behaviours in teens. Journal of Adolescent Health.	USA	2002	n=223	12-18	Health
	Jaya PH, et al. Differences in young people’s reports of sexual behaviours according to interview methodology: A randomized trial in India. American Journal of Public Health.	USA	2008	n=1293	15-19	Health
	Kauer SD et al. Investigating the utility of mobile phones for collecting data about adolescent alcohol use and related mood, stress and coping behaviours: Lessons and recommendation. Drug and Alcohol Review.	Australia	2009	n=18	14-17	Health
**Audio diary**	Sargeant S, Gross H. Young people learning to live with inflammatory bowel disease: Working with an ‘unclosed’ diary. Qual Health Research.	UK	2011	n=6	11-16	Health
**Art**	Coad J et al. Involving children and young people in the development of art-based research tools. Nurse Researcher.	UK	2009	n/a	11-18	Health
	Coad J. Using art-based techniques in engaging children and young people in health care consultations and/or research. Journal of Research in Nursing.	UK	2007	n/a	Not specified (discussion paper)	Health
	Di Gallo A. Drawing as a means of communication at the initial interview with children with cancer. Journal of Child Psychotherapy.	Switzerland	2001	n/a	Not specified (discussion paper)	Psychology

**Table 3:** **Summary of strengths and limitations of data collection method**

**Data collection methods**	**Strengths**	**Limitations**
**Digital technologies**	• Young people generally engaged in the digital world. Sense of immediacy 24 hour access. Researchers can ‘access potential participants’ via ‘chain of friends’. Can give voice to marginalised groups (excluded young people). Web based data collection can provide greater anonymity. Good for sensitive topics. Cheaper than more conventional methods e.g. posting questionnaires/interviews. Minimise stigma Open responses May improve response rates	• Over-disclosure. Vulnerable to cyber-bullying or security problems. May exclude those from poorer socio-economic backgrounds. Harder to establish rapport over the internet/e-mail
**Focus Groups**	• Group setting can prompt debate and discussion. Generates large amount of data	• May not be useful for exploring sensitive issues May feel intimidating for some. Need to consider the mix (gender, sex, age)
**Questionnaires**	• May be completed in privacy, over a period of time. Can be sent to large numbers of potential participants. Can gather large quantities of data	• Good for quantitative data collection, less so for qualitative data collection. Expensive (postage/administration)
**Interviews**	• Promotes honest responses. Establishing rapport. Researcher can read non-verbal cues Opportunity to probe and explore responses	• Social acceptability bias. Power differentials may inhibit responses Cultural and gender differences
**Telephone**	• May be better for sensitive data collection. Access of young people via mobile phones	• May be more difficult to interpret In longitudinal research, may increase drop-out rate if phones are lost/numbers changed
**Audio diary/diaries**	• Can record thoughts/feeling as and when. Freedom of expression. Audio diaries can capture the immediacy of experiences and thoughts	• Paper diaries may feel too much like homework Paper diaries dependent upon literacy skills. Confidentiality
**Arts-based methods**	• Useful to aid expression. Useful way of capturing attention and establishing rapport It can be fun. Good for exploring sensitive topics	• Older adolescents may find it patronising. May benefit from use with other methods. Intimidating if participants lack confidence in creative abilities.

### Papers reporting an overview of methods

Walker’s paper [20] discusses the types of methodologies for data collection with children and young people and notes that the use of vignettes can be particularly useful as ‘ice-breakers’ in this population.

The author reports that the use of stories with a strong moral opinion can start discussions that are more confident and can provoke debate and interest for the participants. The use of pictures and photos are also useful for capturing attention as can the use of well known quotations to strike up discussions. Linking these with popular culture can also harness a young person’s interest and encourage engagement in the topic area. The authors note that researching with children and young people requires reassurance that there are no wrong answers.

One way of demonstrating this in an individual interview setting is to use an ‘about me’ sheet to remind participant that they are the focus of the interview and that their opinions count. It can help reinforce the notion that there are no ‘right’ answers.

Focus groups when conducted with this group can encourage individuals to voice their opinion when others do. The other ‘voices’ in the group can stimulate, challenge and prompt individuals to express their thoughts, feelings and opinions. The authors do note that not all children and young people find this a useful forum. In terms of undertaking mixed sex focus groups, the authors note that at certain ages gender differences can stimulate debate and different styles of expression.

Fargas-Malet and colleagues [21] paper discusses the methodological issues of researching with children and in particular, discusses the innovative methods that are being used in research with this group.

As mentioned in the previous paper, the use of visual aids, for example photos can act as useful prompts for discussion in interviews. Their use can help maintain rapport between the child and interviewer and can provide a focus and elicit information. The authors suggest that this can lead to a ‘deeper understanding’ than a simple conversation. If children or young people are tasked with taking photographs as a means of expression or storytelling, it is important that issues of confidentiality are carefully considered

Drawings can also be a fun way to enable children and young people to express views and experiences. The authors note that it is important to focus not just on drawing, but on what children say about drawing. This can be an efficient way of obtaining large amounts of detail. This technique may be best used in conjunction with other data collection methods as it can be difficult to analyse data from drawings alone. However, the evidence for the efficacy of utilising art and diaries as means of data collection are well described. They can enhance the conceptualisation of knowledge and experiences and their interpretation can add to the knowledge base around the topic explored [22,23].

Q methodology [24] involves ranking of preferences, but is not restricted to ‘written’ words, but pictures, computer generated images, or foods. It has the advantage that it can be delivered face to face, via post, or using web based programmes.

The authors report that the use of a diary or other narrative techniques can be a useful way to explore children and young peoples use and perception of time. However, they raise the issue that such a technique can feel to akin to school work and therefore, may put off some children or young people. Again, issues of confidentiality must be carefully considered as the participants may be using the diary to record very personal and intimate information.

Finally, the authors discuss the relatively common form of data collection - the questionnaire - can be adapted using a format that is more amenable for use with children and young people. For example a recorded questionnaire may be played on personal stereo with the answers written in booklet. Using the telephone of a computer to collection questionnaire data has the added advantage of being relatively quick to administer and to collect large amounts of data. For some young people, answering questions, particularly on sensitive topics, may be much easier to do in private rather than in a face to face setting. Of course, literacy levels must be considered in using questionnaires as a research technique and for some young people and children this method would not be appropriate [25].

The methodological strategies employed across seven studies with children and adolescents are outlined in Christian et al’s paper [26] with the aim of illustrating developmentally appropriate, creative strategies with children and adolescents in a research setting. These methods were qualitative, quantitative and mixed methods and include individual interviews, group interviews, creative thinking, creative play and the use of incentives. The use of these research techniques were all found to actively engage the participants. The authors note that engagement was enhanced by using age appropriate techniques, for example, using game playing to examine the attitudes of younger children towards health related issues.

### Digital technologies

Murthy presents a critical examination of the use of digital technologies in ethnographic research citing the recent upsurge in the use and sophistication of these technologies as a prompt for their use in the filed of social research [27]. Although not specifically considered in terms of their use with CTYPs, the authors report that the nature of some of these technologies e.g. blogs and networking sites make these particularly amenable for use with a younger demographic.

The authors note that on-line questionnaires have the advantage of being cheaper, easier to store and to analyse, but more importantly, it is noted that they can often elicit ‘richer’ responses, especially when the topic is of a sensitive nature. They provide a more powerful sense of anonymity when compared with data collected in a ‘face-to-face’ setting. On-line data collection may also provide a more representative sample, engaging those who would ordinarily decline engaging with research.

The use of digital video has also been utilised in a project at a children’s hospital, where patients are encouraged to ‘teach your clinician about your illness’, in this case, asthma, by uploading video diaries. The authors note that the findings from this project demonstrated not only the participants’ desire to communicate, but their eagerness to communicate the ‘intimate details’ of how they experienced their illness. The proliferation of webcams in the work and domestic environment makes this tool another useful data collection method. It is noted that responses via a webcam may be less ‘staged’ than those elicited via video diary, as the camera is much smaller and less imposing then those traditionally used in this sphere.

Social networking sites for research purposes can be both useful, but also to be used cautiously. They can enable researchers to access ‘chains of friends’, akin to the use of snowball sampling [28] to recruit participants. They can also provide access to more marginalised groups, the voices of whom are seldom heard. However, the authors note that despite their allure, membership to on-line communities can be restricted to the ‘haves’ who are able to access and manipulate such networks and the ‘have nots.’ The authors note that the use of social networking sites for research should be considered alongside other methods rather than as a stand alone technique.

It critically examines possible problems of technologies.

The use of blogs has been useful in giving a voice to those who are ‘traditionally unrepresentated’, and can be seen as a ‘potentially democratizing force’, although as with social networking and other traditional ‘off-line’ public spheres, where ‘patriarchal hegemony persists’.

The authors conclude that despite the exciting array of novel methods that can be harnessed in the research world, they should not replace conventional methods, especially as access to these technologies is still dependent upon class, race and gender issues.

Cranmer’s study focuses upon young people who have been excluded from mainstream education and how the use of digital technologies can enhance or challenge their lives [29]. In-depth interviews were conducted with 13 young people, and although the study is not focused upon using digital technologies to collect the research data, it provides a nuanced picture of how such technologies are used by this group. The young people interviewed used these technologies for entertainment and keeping in touch with friends and family. Given that these young people have been excluded from school and are held in a ‘participation gap’ the usefulness of this as a communication tool is clear. In some ways, children who undergoing treatment for cancer will also experience a ‘participation gap’ as they may be unable to attend their usual school for long periods and feel socially isolated from their peers. The authors urge some caution in terms of the use of digital technologies as they can leave vulnerable young people exposed to ‘unsafe’ on-line material. However the benefits for this already marginalised group are clear.

Baer et al. undertook a prospective cohort study to estimate the transmission rates of Human Papilloma Virus (HPV) in young adults (women age 18-20) and describes design methods and implementation issues [30]. The paper provides a useful overview of the advantages of web based data collection including:

- It can provide greater anonymity, especially if a sensitive subject is being explored, than face to face interview and may yield more truthful answers.
- The participant can choose time and place to complete
- Data can be returned to researcher much quicker
- It is a cheaper means of conducting research compared with printing and posting questionnaires.

The study asked participants to complete a sex behaviour questionnaire and web based diary entry and found that the sense of privacy that accompanies on-line diaries was of benefit given the sensitive nature of the subject matter.

As the data was collected at short intervals, recall bias was also reduced.

The authors report that this method is not without obstacles. A level of expertise is required in setting up software and security/confidentiality issues have to be carefully considered. Furthermore, this method may exclude those without access to the internet so they may not be representative of the population.

A study focusing upon the feasibility of e-mail communication for interviewing young people with mental health problems (Cleary, Walter) also provides a useful summary of the advantages and disadvantages to this mode of data collection [31]. The authors report that it allows participants a longer period of time to reflect on questions, compose answers and respond at leisure in comfort. It may enhance participant autonomy, and can be conducted over longer time periods. As noted in Baers, it is also more cost effective (printing/posting/travel expenses are not required) and means those living far from the research site can access just as easily as those in closer geographical proximity [30].

The authors note that TYP’s tend to be ‘savvy’, when it comes to using digital technologies and may find this method less intrusive than face to face interviews. It can provide greater privacy, minimise feelings of stigma, and hence yield more ‘open’ responses.

However, the authors further note that e-mail communication limits the ability to establish a rapport between the researcher and participant, which when addressing sensitive issues can in itself help to create an atmosphere of trust and openness.

Technical problems can also arise, which can compromise the confidentiality and anonymity of participants. Furthermore, as e-mailing requires literacy skills that not all participants may possess, this method may exclude those who struggle to express themselves in the written form.

Overall, the authors recommend that further work is undertaken to examine the acceptability and utility of using e-technology to undertake research.

A further example of using an on-line forum was focused upon children and young people undergoing active treatment for cancer [32]. In this study, the forum was set up as an on-line focus group and the authors conclude that the level of anonymity that this forum gave participants made it much easier for them to comfortably express themselves. Participants also valued the flexibility and convenience of logging in at their own time and place to join discussions.

Other examples of studies using various digital technologies include a paper focused upon the use of an Interactive Voice Response (IVR). This device was also found to be a feasible and relatively anonymous method of data collection when trialled for use with adolescents who had been the victims of assault [33].

Des Jarlais and colleagues’ study attempted to keep mobile drug users engaged in services by issuing electronic debit cards which paid out after they phoned in to study to complete interviews [34]. The study found that combined with other efforts to develop positive relationships, the use of electronic cards led to higher participation than noted in previous studies.

The use of audio-enhanced personal digital assistants as a method for data collection was also shown to be feasible for use with school students, keeping students engaged in the research process and improving response rates. It was also found to be effective for students with cognitive impairments or language barriers, who previously struggled with more formal modes of data collection [35].

Sargeant and colleagues explored the use of an audio diary as a means to record their experiences of young people living with chronic disease [36]. The authors found that the diary revealed the ‘ordinariness’ of their experience. The method provided immediacy and intimacy, conversation and reflection as well as flexible method of recording experiences.

Denny and colleagues reported the findings from a study comparing adolescence preference between hand-held internet tablets and the use of a lap-top in administering health and well-being questionnaires [37]. The findings of this study were relatively inconclusive with many participants expressing no preference between two modes. For those who did express a preference the majority of students found the hand-held tablet more private and confidential than using a lap-top.

McCabe and colleagues examine the efficacy of using a web-based survey to collect alcohol and drug data with young people in USA. The study uses a large and ethnically diverse sample [38].

The authors point out that due to financial constraints and the emergence of digital technology, there is a need to look beyond postal surveys as the sole mode of data collection. The study does not compare postal and web-based methods but instead it aims to examine the feasibility of implementing a web-based survey approach to collect data in a racially and economically diverse population. The authors report a high response rate - 89%, although they note that the response rate decreased with age. As seen with Baers and Cleary [30,31], the authors note the advantages of using this method in terms of the good response rate; a fast turnaround in terms of data collection; it yielded high data quality in a potentially sensitive subject area and it was cheaper to administer than more conventional methods.

### Arts-based research

The benefits of using art as a method of data generation and engagement with young people are described in three papers. Di Gallo and colleagues’ paper focuses on the use of art as a way of enhancing communication around the issue of cancer [39]. The authors assert that you cannot always build a trusting or meaningful dialogue with words alone and that drawings may enable children to express feelings without ‘giving up resistance’ i.e. they may still not want to disclose completely due to the nature of distress the they are experiencing.

The need for greater participation of children and young people in research is expounded by Coad and art can be a rewarding and challenging method of engaging with this group; it is a ’powerful medium through which children can express their views across a wide range of developmental continuim’ [40]. Coad provides some useful tips in terms of balancing the ‘power’ between researcher and participant.

- Adopt role of naïve curiosity - open, honest and understanding but not patronising.
- Avoid being judgemental, but accept child’s view as different to adults.
- Allow child to present views
- Be creative and flexible to reduce boredom.

Coad and colleagues note that arts-based techniques can be particularly useful when seeking to explore sensitive issues, in this case to assist with familial discussions around rare genetic conditions [41].

### Focus groups

Banister et al report the findings from a focus group aimed at investigating adolescent women’s health concerns pertaining to relationships (14-19) [42]. This appeared to be a particularly effective way of data-collection for this group as it was able to harness shared experiences, collective knowledge and expertise between group members. The group setting, the authors note, is congruent with the way women in Western culture have been socialised to understand, communicate and construct meaning lending complexity and richness to data and ‘provides a microcosm of the very social and relational interactions we intended to study.’

Emphasis was also placed upon participants seeing themselves as co-researchers’ thus challenging the power imbalance that can arise in a research setting. The groups ran over a period of 18 months, sometimes just ‘hanging out’ in the local community centre, and this informality seemed to appeal to the particular age range.

Yonekura and colleagues used the forum of a focus group to engage young people in the topic of ‘youth values’ , but also introduced a ‘game’ element to the proceedings [43]. Participants had to pick out a statement from a bag and attach it to a piece of cardboard labelled with a 5 point likert scale in terms of levels of agreement or disagreement with the statement. Discussions around the choices they made were encouraged and the authors found that the young people valued this aspect of the interaction. It appeared to enhance expression and discussion. The young people felt ‘involved’ and it appeared to harness a respect for any differing opinions across the group.

### ‘Paper and pencil’ versus computer

Five papers compared the use of a web-based computerized method of data collection with the more traditional ‘pen and paper’ mode of administration.

As part of a study comparing health questionnaire scores obtained via the internet versus scores obtained via ‘pen and paper’ Mangunkusumo and colleagues found that participants using the internet method for completion rated this method more favourably than those who undertook the ‘pen and paper’ version [44]. The internet version also took less time to complete, thus reducing participant burden. Scott-Johnson and colleagues assessed response rates to a health questionnaire depending upon method of administration (web-based vs paper surveys) [45]. They also found that the web-based method had a better response rate than the ‘pen and paper’ method. Wu and colleagues found similar response rates in terms of the mode of administration, but there was a higher level of incompleteness in the ‘pen and paper’ version, suggesting that web-based administration with adolescence may be more amenable to health related research in this population [46].

By constrast, two studies comparing the use of the web-based data collection with the ‘pen and pencil’ found a preference for using the more traditional mode of completion. Beebe and colleagues’ study aimed to see how information obtained from an adolescent screening instrument administered in a medical clinic was affected by the data collection methods used [47]. Participants were asked questions around mental health, sexual experiences, and substance use. The authors found that participants reporting of risk behaviour was higher on the paper than the computer version, suggesting that respondents felt more comfortable answering questions of a sensitive nature via this method. Wyrick and colleagues compared the bias between web-based and paper and pencil responses to questions related to substance use, again a sensitive subject [48]. Similarly, this study found higher responses in the ‘pen and paper’ mode of administration. They found that respondents were more likely to skip questions on the web-based mode of administration, despite the sense that this is the more ‘private’ method.

### Questionnaires/interviews

Plummer et al. examined the validity of data collected in relation to sexual behaviour of African adolescents [49]. The authors found that in-depth interviews appeared to be more effective than self-completion questionnaire and face-to-face questionnaires in promoting honest responses, particularly for female participants. It is plausible that an in-depth interview enables a more nuanced approach to be taken when investigating a sensitive subject like sexuality. Dockrell and colleagues explored the methodological issues when researching with young people around sexual behaviour and perceived risks of contracting HIV and the authors conclude that using multiple modes of assessment (for example questionnaires and open-ended interviews) can be the best approach to gaining robust data [50]. It is noted that using both modes of administration enables researchers to explore some of the apparent contradictory responses about perceptions of ‘risk’ behaviours.

Kann et al’s study aimed to examine the effect of data collection setting on the prevalence of priority health risk behaviours among adolescents [51]. The authors compared a school based survey with a household survey. The school based survey produced higher prevalence rates than the household surveys and this is accounted for by the increased feelings of privacy, namely privacy from parents, felt in the school. The authors also discuss the advantages and disadvantages of each mode of administration. The school based survey is less expensive and more efficient and can provide young people with an enhanced sense of privacy when compared to the household survey. However, the household survey can gather more information as its completion is not limited by lesson times. It can also provide access to young people not engaged in full-time education.

Jaya and colleagues’ study aimed to compare the reports of sexual behaviours given in standard face-to-face interviews with reports given in audio computer assisted self-interviews (ACASIs) and culturally specific interactive interviewing among adolescents in India (aged 15-19) [52].

The study found that the reporting of sexual behaviours differed according to interview methodology. Boys and girls both reported more sexual behaviours in the interactive interviews than in face-to-face and the authors conclude that this may be explained by the cultural specificity of the, enabling greater comprehension of the subject matter and research questions.

### Telephone

Ellen and colleagues compared the response bias associated with telephone survey or in-home self-administered audio computer assisted interview in terms of STD/HIV related risk behaviours [53]. The population were African American adolescents. No differences were observed in terms of perceived comfort, honesty or accuracy in answering questions across the different modes. However, the authors conclude that the telephone, the most economical mode of administration, can be employed without much risk of increasing response bias in terms of assessing risk behaviours.

Kauer and colleagues investigated the utility of using a mobile phone for collecting data about the alcohol use and related mood, stress and coping behaviours of younger adolescent who attended school compared with older adolescents who were working or in tertiary study [54]. The authors found that mobile phones captured larger amounts and higher quality data, thus suggesting that the mobile phone is an acceptable form of data collection with younger adults. This mode of data collection was found to be particularly useful in capturing data on a daily basis.

This review aimed to explore the range of data collection methods employed to undertake research with CTYPs. It is of note that the majority of studies focused around the utility of collecting data of a sensitive nature whether it be around sexual behaviours and HIV (Wyrick, Plummer, Dockrell, Ellen, Jaya), drug and alcohol use (Des Jarlais, McCabe, Kauer), mental health problems (Cleary), health behaviours (Yonekura) and experience of living with cancer (Tates and DiGallo). The sensitive nature of these topic areas along with the associated issues of adolescence would appear to indicate that methods of data collection that are most amenable and appealing to CTYPs should be employed if researchers are to generate meaningful data around these potentially ‘difficult’ topics. As stated in the study background, a diagnosis of cancer and the effects of treatment can have a particularly profound effect upon young people. This review aimed to identify the range and utility of data collection methods used for research with young people with a view to undertaking further work to ascertain from young people with cancer how they would most prefer to communicate with researchers about their experience.

Papers were identified if they had a particular focus upon the utility of the methods used. Although a comparatively large number of studies have been undertaken focusing upon CTYPs, the majority of these identified in the initial searches focused upon the particular study outcomes rather than upon the particular data collection methods employed.

This review presents the findings from a systematic search of the literature pertaining to the data collection methods employed with CTYPs and to provide a narrative summary of the findings from the studies identified.

Due to the heterogeneity of the studies included, the results presented the papers in line with the broad category of the data collection method employed.

Walker and Fargas-Malet both recommend the use of ‘prompts’ for discussion when working with CTYPs [19,20]. These prompts may be visual, for example, the use of pictures or drawings, or auditory prompts such as stories, or presenting controversial statements to provoke debate and discussion of a topic.

The largest proportion of included papers focused upon the use of digital technologies for research with the population of interest, demonstrating their scope or use and the advantages and disadvantages of their use.

For example, on-line questionnaires can be a more cost-effective method of acquiring large amounts of data [23,26,27] and a greater sense of anonymity encouraging participants to feel comfortable to disclose their experiences [23,26]. They can also provide a more representative sample of participants, reaching out to those who may not ordinarily engage in research activity, or who are excluded from mainstream education [25]. This method of data collection may be particularly useful when exploring subjects of a sensitive nature [26,34]. This makes them particularly amenable for use with CTYPs where the power imbalance between researcher and participant may be more pronounced than between researcher and adult participants. Disclosing sensitive information, for example around sexual behaviours, drug or alcohol use or mental health problems, in a face-to-face setting may prove challenging for a young person who is undergoing the emotional and cognitive challenges that can accompany adolescence [10,11]. Tates et al’s study reported the usefulness of an on-line forum for young people, in this case young people undergoing cancer treatment. The level of anonymity it provided enabled the participants to feel they had a ‘safe’ platform for disclosure about their fear, anxieties and experiences.

However, as the authors note there can be inherent difficulties in utilising digital technologies for research such as technical difficulties related to software used and the extra caution required in terms of maintaining confidentiality and anonymity. This method may also exclude CTYPs who have limited literacy skills or access to a computer, thus reinforcing inequalities.

Three studies focused upon comparing web-based data collection methods with the more traditional method of ‘pen and paper’ and found that the web-based format had a better response and completion rate [40-42]. Conversely, two studies comparing these two methods found that the ‘pen and paper’ method elicited higher levels of disclosure when exploring sensitive topics [43,44].

Despite these equivocal findings, there is clearly a place for web-based technologies, given their even burgeoning availability and sophistication.

Murthy at al espouses the use of social networking and blogs as a mean of accessing, communicating and ‘researching’ with CTYPs, especially given their general proliferation amongst the younger generation. Cleary et al also note that CTYPs tend to be more ‘savvy’ in their manipulation of web-based technologies.

However, Murthy indicates that social networking as a way of undertaking research has its limitations. Despite being able to engage with apparently marginalised groups, it may be more difficult to access a representative sample, which is not an issue for undertaking qualitative research, but may impact upon the results from quantitative studies. The author recommends their use in conjunction with more traditional techniques.

The use of video or web-cam diaries are further cited as suitable method of data collection. When utilised in a paediatric setting to encourage patients to record the ‘lived experience’ of their illness to send to their clinicians, Murthy notes that the participants felt comfortable in presenting a detailed and intimate insight into the nature and manifestation of their illness [23]. An on-line diary can also reveal the ‘ordinariness’ of everyday experiences of living with a chronic disease [32].

Other modes of digital technology; mobile phones, audio-enhanced digital assistants and interactive voice response devices were also deemed to be an effective data collection method with this population.

The use of art-based techniques to engage CTYPs in research is shown to enhance the ability for researcher and participant to communicate [35] and encourage the expression of the thought, feelings and experiences of the group. The use of art in the research setting can be challenging, but ultimately fruitful and rewarding, enabling a ‘dialogue’ to be expressed though the medium. It is further noted that this method can generate high quality data, especially when the topic of interest is of a sensitive nature [36,37].

The relatively traditional modes of data collection - focus groups, interviews and questionnaires - can also be utilised in research with CTYPs.

Group discussions can be an appropriate forum to encourage debate and generate ideas. Those taking part can feel they are part of the research process and thus will invest in the sessions. They are also useful in terms of generating relatively large quantities of qualitative data.

Although the use of remote means of data collection (web-based, on-line forums) provide a greater sense of anonymity, encouraging honesty and high levels of disclosure, it is also noted that face-to-face in-depth interviews have the advantage of enabling the researcher to probe participants, and check understanding of the responses and allowing the participant to clarify any points they may make. It could be argued that in this sense, they are able to provide data that enables a more nuanced picture of the phenomena being investigated. Dockrell et al suggest providing a mixed methods approach, using both questionnaires in conjunction with open-ended interviews, can produce high quality, robust data [46].

None of the included papers examined the credibility of the data collected in terms of the particular method employed. For example, none of the papers compared data collected from a focus group setting with the data collected from an on-line forum. As touched upon, it is conceivable that given the sensitive nature of a particular topic, for example exploration of sex and sexual behaviours it is plausible that certain collection methods would invite a more ‘honest’ response. For example, data collected on this topic from a focus group may be inhibited by the group setting. Participants may feel that they may be judged by other group members and their may be a degree of social acceptability bias in the data collected. This may be particularly pronounced amongst a group of young people. Using anonymous on-line methods of data collection are likely to elicit more candour. This issue of the credibility of data gathered via different collection methods is an important and warrants further investigation.

## Conclusion

In conclusion, there are a number of data-collection methods, both novel and traditional that can be utilised to generate data when working with CTYPs. They each have their own strengths and limitations as their utility may depend upon such factors as the age range of participants - for example, arts-based methods may be more appropriate to working with a younger age group, and focus groups or digital technologies may be preferable when working with middle and late adolescents.

Given the breadth of data collection methods utilised with CTYP, the next stage of this study will survey teenagers and young people with cancer.

Assessing the available evidence will help inform the next stage of the study which will focus upon teenagers and young people who are living with cancer to ascertain the preferred data collection method with this particular population.
